# Improvements in the Production of Isosorbide Monomethacrylate Using a Biobased Catalyst and Liquid–Liquid Extraction Isolation for Modifications of Oil-Based Resins

**DOI:** 10.1021/acsomega.4c01275

**Published:** 2024-05-31

**Authors:** Vojtěch Jašek, Jan Fučík, Veronika Melčová, Radek Přikryl, Silvestr Figalla

**Affiliations:** † Institute of Materials Chemistry, Faculty of Chemistry, Brno University of Technology, Brno 61200, Czech Republic; ‡ Institute of Environmental Chemistry, Faculty of Chemistry, Brno University of Technology, Brno 61200, Czech Republic

## Abstract

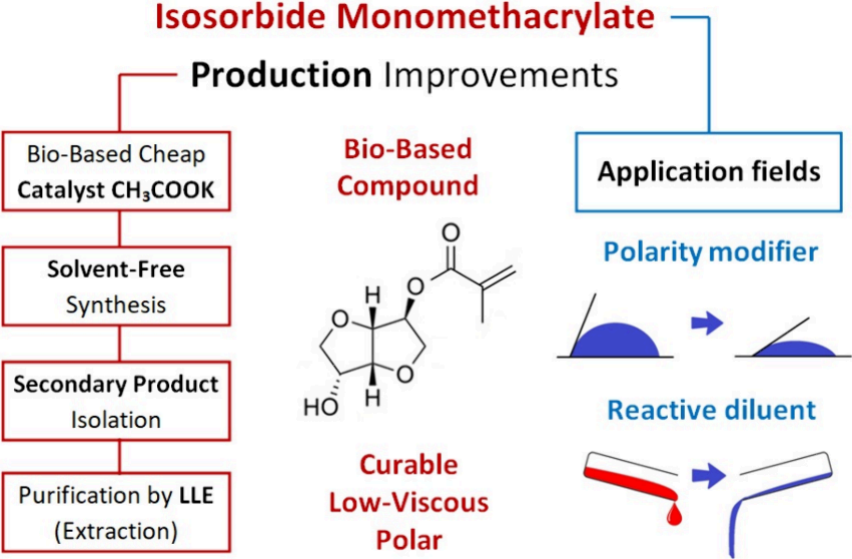

The improved production of a polar curable monomer, isosorbide monomethacrylate (MISD), with methacrylic anhydride (MAAH) as an acyl donor, was performed. A sustainable and cheap catalyst, potassium acetate (CH_3_COOK), was used for a solvent-free synthesis, requiring only the equimolar amount of reagents (no excess). The production included the quantitative separation of the secondary product, methacrylic acid (MAA), preventing the reaction batch from the purification process (neutralization of MAA), and gaining a usable reagent. The synthesis resulted in a sufficient yield of MISD (61.8%) obtained by the liquid–liquid extraction process (LLE), which is a significant improvement in the process, avoiding the flash chromatography step in the isolation of MISD. The purity of synthesized and isolated MISD via the LLE was confirmed by ^1^H NMR, MS, and FTIR analyses. The thermal analyses, namely, DSC and TGA, were used to characterize the curability and thermal stability of MISD. The activation energy of MISD’s curing was calculated (*E*_a_ = 94.6 kJ/mol) along with the heat-resistant index (*T*_s_ = 136.8). The polar character of isosorbide monomethacrylate was investigated in a mixture with epoxidized acrylated soybean oil (EASO). It was found that MISD is entirely soluble in EASO and can modify the rheological behavior and surface energy of EASO-based resins. The apparent viscosity of EASO at 30 °C (η_app_ = 3413 mPa·s) decreased with the 50% content of MISD significantly (η_app_ = 500 mPa·s), and the free surface energy value of EASO (γ_S_ = 42.2 mJ/m^2^) also increased with the 50% content of MISD (γ_S_ = 48.7 mJ/m^2^). The produced MISD can be successfully used as a diluent and the polarity modifier of curable oil-based resins.

## 1 Introduction

Sustainable structures synthesized from renewable sources that can be cured have recently attracted vast attention. Reactive molecular systems serving as precursors of stiff and rigid or elastic and flexible materials can fulfill their purposes in numerous applications such as 3D printing matrices,^1−3^ transparent supportive layers in the electronic field of uses,^4^ functionally permeable^5^ or mechanically protective coatings,^6,7^ thermosets for flame retardants, and others.^8^ The sustainability of potentially produced materials is critical, but working with low-cost available entering substances is essential to ensure an economic rentable factor. Various thermoset precursors can be mentioned providing named characteristics such as modified (epoxidized, acrylated, or methacrylated) vegetable oils;^9−11^ curable derivates of standard polyols (ethylene glycol, glycerol, erythritol, sorbitol)^12−15^ or other biobased structures able to be modified to reactive products (vanillin, ascorbic acid, alkyl carboxylates).^16−18^ All appointed representatives fulfill the natural origin, but particular applications of curable precursors require specific physical–chemical properties such as appropriate rheological behavior, sufficient material strength/flexibility, or predictable surface tension and surface energy of formed cured resins. Suitable viscosity levels are essential for all coating systems due to the varying nature of treated substrates and the very thin layering requirements that can be formed most efficiently from precursors possessing low-viscosity values.^19,20^ Mechanical protective applications depend upon appropriate elastic modulus values and glass transition temperatures of formed thermosets since their rigidity/flexibility is the key exhibited property.^21,22^ The adhesion and permeability are primarily dependent on the surface tension and energy of curable precursors and formed resins since the overall interaction with the surroundings, such as swelling or diffusion, is influenced by the number of molecular interactions of either nonpolar or polar attraction forces.^23−26^

Isosorbide (1,4:3:6-dianhydro-d-glucitol) is a C_6_ compound with two furan-like cycles and two available hydroxyl functional groups.^27^ This diol can be synthesized via various pathways, from which the dehydration of sorbitol is the most well-known and described.^28^ Different cellulose polymeric compounds and other glucose-containing structures are used to produce isosorbide because glucose can be reduced to sorbitol via numerous catalytic routes.^29,30^ Isosorbide is a crystalline compound that can be used to prepare polyesters of polyurethanes thanks to the presence of two hydroxyl functional groups.^31^ Along with the direct incorporation of isosorbide in the mentioned polymeric structures, this diol can also be modified, leading to the formation of nitrates,^32^ esters,^33^ ethers,^34^ and other derivatives of the original molecule. The exceptional rigidity of products containing isosorbide or its derivatives is a connecting property of many described materials. The brittleness of such structures illustrates the glass transition temperature of particular resins and polymers (isosorbide dimethacrylate *T*_g_ = 142.2 °C;^35^ acetylated methacrylic isosorbide *T*_g_ ≈ 130 °C;^36^ and modified diphenol-terminated isosorbide *T*_g_ = 143–193 °C^37^). Due to these properties, isosorbide is often used as an alternative to structures containing bisphenol A (a molecule with two available hydroxyl groups exhibiting high rigidity and brittleness).^38^ Also, due to the low molecular weight of isosorbide and its various derivatives along with the unique structure composition resulting in minimizing of hydrogen bonding and other intermolecular attractive forces of such compounds, the viscosity levels of these structures are usually deficient, which can be beneficial not only for specific applications but for the potential determination of final rheological profile of resin mixtures.^39^ The viscosity adjustments using isosorbide derivatives were investigated (isosorbide/styrene mixture viscosity η_25°C_ = 5 ± 1 cP compared to Derakane 441–400/styrene mixture (same ratio) viscosity η_25°C_ = 200 ± 40 cP)^40^ combining the cross-linking properties and an appropriate rheological behavior.^41^

Cutting-edge resin precursors based on isosorbide as a renewable source are isosorbide monomethacrylate. Due to an acylation reaction with the present hydroxyl, this compound contains one methacrylate functional group. The other OH-group remains vacant, which ensures particular properties regarding the potential modification of surface tension and energy of forming resins.^42^ Specific synthesis pathways were investigated and described in the literature using different acyl donors such as acyl halides,^43^ anhydrides,^44^ esters,^45^ or carboxylic acids.^46^ Acyl halides are the most reactive donors; however, the production scale-up is inefficient due to the nature of the reaction process. Vinyl methacrylate as a particular ester was also used for the synthesis of isosorbide monomethacrylate resulting in high-yield production (87% yield)^47^; nevertheless, the regeneration of allyl alcohol as a secondary product was not considered besides the fact that the reaction includes enzymatic catalyst (*Rhizomucor miehei* lipase) which is an expensive component.^47^ Other acyl donors (anhydride and carboxylic acid) resulted in low yields in other investigated cases (from 17 to 40% in different studies).^48,49^ Furthermore, the suggestion of secondary product usage (formed allyl alcohol or methacrylic acid) has not yet been published. These compounds can complicate the production process since it is essential to dispose of them with concern for the environment.^50,51^ Isosorbide monomethacrylate has a solid potential to serve purposes such as polarity modification, enrichment of the biobased content in curable mixtures, enhancement of the mechanical properties due to its unique molecular structure, or the determination of the rheological profile of formed systems containing it.

**Figure 1:**
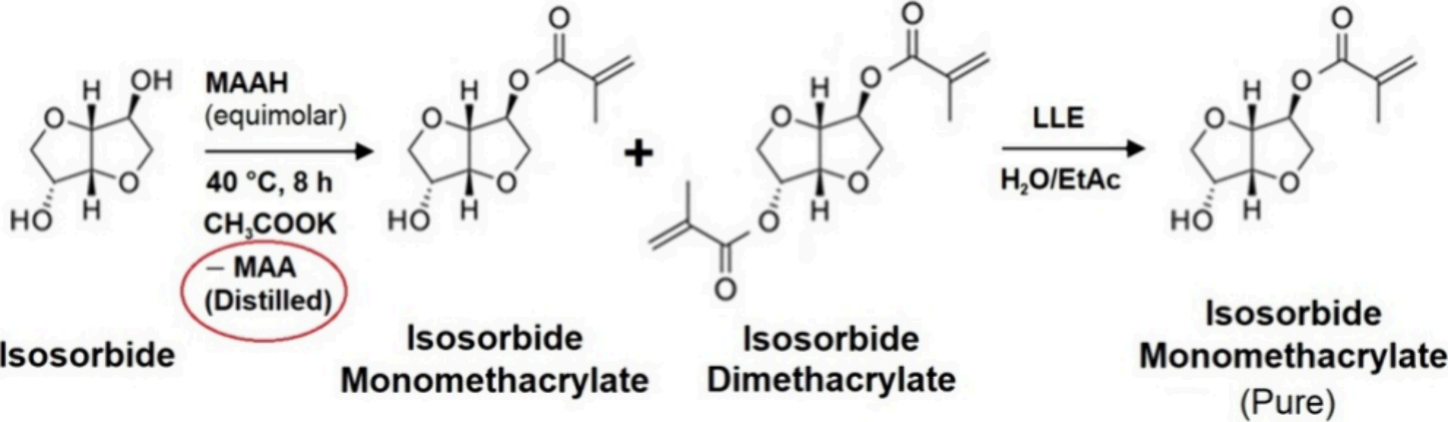
Reaction and purification scheme of isosorbide monomethacrylate (MISD) synthesis via catalysis with potassium acetate (CH_3_COOK) and liquid–liquid extraction (LLE).

**Figure 2:**
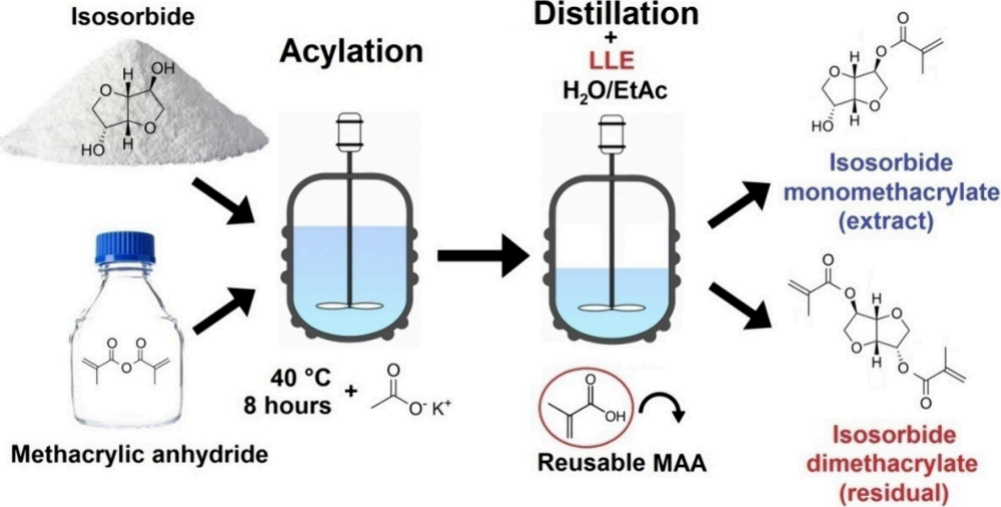
Production of isosorbide monomethacrylate via acylation with methacrylic anhydride as the acyl donor and potassium acetate as the catalyst using liquid–liquid extraction (LLE) purification and distillation separation of the secondary product methacrylic acid (MAA).

This presented article describes a complete production pathway of isosorbide monomethacrylate (Figures 1 and 2) using methacrylic anhydride as an acyl donor and a low-cost and sustainable catalyst, namely, potassium acetate (CH_3_COOK). Moreover, the presented production process includes the possibility of scale-up because no reaction solvent was used compared to the proposed investigations, and the formed secondary product, methacrylic acid, was separated from the reaction mixture via distillation. The purification process that we describe involves isolation via liquid–liquid extraction. The synthesized products were structurally verified via numerous analytical methods, such as ^1^H NMR, MS, or FTIR. Thermal analyses were performed on the produced compounds, particularly DSC, to verify the reactivity of the structures and TGA to measure the heat-resistant indexes of cured resins. Additionally, the solubility of synthesized isosorbide esters was studied since modifications in the resins’ properties are considered along with the measurement of the rheology of the precursors. Eventually, the polarity adjustment effect of the isosorbide monomethacrylate was explored via the contact angle measurement of prepared resins containing this polar molecule.

## 2 Experimental Section

### 2.1 Materials

Isosorbide (1,4:3,6-dianhydro-d-glucitol; 99%) was kindly acquired from Novaphene, India. The extraction solvent ethyl acetate (EtAc, 99%) was purchased from Penta Chemicals, Czech Republic. The anaerobic inhibitor of spontaneous polymerization, used during the distillation of methacrylic acid from the reaction mixture, Genorad 26, was bought from UL Solutions, Northbrook, IL, USA. The catalyst for acylation (potassium acetate, 99%), the acyl donor methacrylic anhydride (MAAH, 94%), BAPO initiator for the photoinitiation (phenylbis(2,4,6-trimethylbenzoyl)phosphine oxide, 97%), Luperox DI for the sample preparation of DSC and TGA analysis samples (*tert*-butyl peroxide, 98%), and epoxidized acrylate soybean oil as a matrix for surface tension and energy modification were all acquired from Sigma-Aldrich.

### 2.2 Structural Characterization

Nuclear magnetic resonance (NMR) served as one of the verifying methods. The measurements (gained via Bruker Avance III 500 MHz (Bruker Billerica, MA, USA)) were performed at 30 °C. The chosen solvent was deuterium chloroform (CDCl_3_) containing tetramethylsilane (TMS) as an internal standard. The acquisition time was 4.0 s. The resulting spectra were described using chemical shift (δ) in ppm with the reference to TMS. Coupling constants *J* (unit (Hz)) are specified: “s” singlet, “d” doublet, “t” triplet, “q” quartet, “p” quintet, and “m” multiplet.

The molecular structure was also confirmed by mass spectrometry (MS) (Bruker EVOQ LC-TQ) using electrospray ionization (ESI). Product scan spectra were obtained by fragmentation of the following [M + H]+ precursor ions: *m*/*z* 215.3 (MISD) and 282.4 (ISDMMA). Collision energy spread (5–20 eV) improved the collected MS/MS data quality. Furthermore, the obtained mass spectra agree with their in silico prediction by CFM-ID 4.0,^52^ which also proposed the product ion structure for the most intensive masses.

Fourier-transform infrared spectroscopy (FTIR) was used to verify the verification methods. We used an infrared spectrometer (Bruker Tensor 27 (Billerica, MA, USA)) measured with the attenuated total reflectance (ATR) method, where a diamond served as a dispersion component. The chosen irradiation source was a diode laser. Michelson interferometer served for the eventual quantification of the measured signal. All illustrated spectra were composed of 32 total scans with a resolution of 2 cm^–1^.

### 2.3 Synthesis and Purification of Isosorbide Monomethacrylate (MISD)

Isosorbide (1 mol, 146.2 g) was transferred to a three-neck bottom flask and heated to 60 °C to melt the reactant. Methacrylic anhydride (1 mol, 154.2 g) was poured into a dripping funnel and placed in the flask with melted isosorbide. When the isosorbide melted, potassium acetate (CH_3_COOK, 0.01 mol, 0.98 g) was added to the flask. Then, the dripping funnel was set to a continual addition of methacrylic anhydride for consecutive 4 h, and the reaction mixture was set to cool to 40 °C. The unreacted isosorbide was dissolving in the forming methacrylic acid. The reaction (see Figure 1) was performed for 8 h until the reaction mixture’s acidity did not increase further. Genorad 26 (3 drops) was added to the mixture, and the system was set to vacuum distillation at 100 °C and 1 kPa until all volatile content was separated. The solution containing isosorbide dimethacrylate (ISDMMA), isosorbide monomethacrylate (MISD), and catalyst (CH_3_COOK) was extracted by water (300 g of water) to separate the water-soluble content (MISD) from the water-insoluble (ISDMMA). Ethyl acetate (300 g) was mixed with water to extract MISD from water. The solution of MISD in ethyl acetate was set to distillation to separate the solvent from the isosorbide monoester. This LLE was performed thrice to obtain all isosorbide monomethacrylate yield (61.8%). ^1^H NMR spectra are shown in the Supporting Information in Figures S1 and S2. The spectral data are summarized as follows:

#### 2.3.1 Isosorbide Dimethacrylate (ISDMMA)

(CDCl_3_, 500 MHz): δ (ppm) 6.12–6.08 (t, *J* = 1.3, 1.3 Hz, 1H); 6.07–6.02 (q, *J* = 1.2, 1.1, 1.1 Hz, 1H); 5.58–5.51 (dp, *J* = 1.7, 1.6, 1.6, 1.6, 1.6, Hz, 2H); 5.22–5.16 (q, *J* = 2.9, 1.2, 1.2 Hz,1H); 5.18–5.11 (q, *J* = 5.5, 5.5, 5.5 Hz, 1H); 4.87–4.81 (t, *J* = 5.1, 5.1 Hz, 1H); 4.50–4.43 (d, *J* = 4.7 Hz, 1H); 4.00–3.77 (m, 4H); 1.95–1.84 (dt, *J* = 20.0, 1.3, 1.3 Hz, 6H).

#### 2.3.2 Isosorbide Monomethacrylate (MISD)

(CDCl_3_, 500 MHz): δ (ppm) 6.17–6.11(dt, *J* = 15.6, 1.3 Hz, 2H); 5.64–5.59(dt, *J* = 3.1, 1.5 Hz, 2H); 5.24–5.18 (m, 1H); 4.92–4.88 (t, *J* = 5.1 Hz, 1H); 4.68–4.61(t, *J* = 4.8 Hz, 1H); 4.57–4.49 (dd, *J* = 4.4, 1.2 Hz, 1H); 4.44–4.38 (dd, *J* = 4.6, 1.1 Hz, 1H); 4.37–3.33 (d, *J* = 3.2 Hz, 1H); 4.21–3.69 (m, 10H); 3.66–3.51 (dd, *J* = 9.5, 6.0 Hz, 1H); 2.68–2.51 (d, *J* = 7.0 Hz, 1H); 1.98–1.94 (m, 6H).

### 2.4 Characterization of Properties

Differential scanning calorimetry (DSC) confirmed the curability of the products. The compounds (ISDMMA, MISD) were mixed with Luperox DI (*tert*-butyl peroxide, 1% (w/w) quantity to product). The solutions were transferred to aluminum pans (6–7 mg) and hermetically sealed. The instrument (DSC 2500 model from TA Instruments (New Castle, DE, USA)) was used for measurements. Four heating ramps were applied on each sample (10–200 °C) with ramps of 5, 10, 15, and 20 °C·min^–1^.

Thermogravimetric analysis (TGA) determined the heat stability index of prepared cured resin precursors (ISDMMA and MISD). The samples used for TGA were the cured resins measured by DSC previously. TGA itself, performed on a TGA Q500 (TA Instruments (New Castle, DE, USA)), also illustrated the degradation process by the particular weight loss temperatures. Two samples in total (ISDMMA (cured), MISD (cured); 5 mg) were measured under the following conditions: equilibration at 40 °C; heating to 600 °C at a heating rate of 10 °C/min under N_2_; heating to 650 °C at a heating rate of 10 °C/min under air atmosphere.

The solubility of MISD was predicted and investigated. Particular RED values (according to Hansen’s solubility theory^53^) were calculated for each compound used in the solubility investigation (the calculation described in Supplementary). According to theory, the solvent is inappropriate for solubilization when the RED value is above 1. The media should be a suitable solvent when RED reaches a value below 1. Used solvents and media for MISD solubility were methacrylic anhydride (MAAH), methacrylic acid (MAA), isosorbide dimethacrylate (ISDMMA), ethanol, ethyl acetate (EtAc), and methacrylated soybean oil (MSO). The calculated RED parameters were experimentally verified by the following method: 3 g of MISD was added into a 3 g particular solvent or compound listed previously. The mixture was mixed vigorously for 5 min and left at laboratory temperature for 1 day. The results of the solubility experiment were illustrated by a photo (see Figure 6).

The rheological behavior was investigated (Brookfield RVDV-II + PX rotational viscometer) to verify the viscosity modification properties of MISD in combination with high-viscous EASO. The apparent viscosity (η_app_) dependency on the temperature increase was measured to calculate Arrhenius parameters.^54^ Samples were prepared as solutions of MISD in EASO (0, 10, 30, and 50% of MISD in EASO). The measurements used a Peltier platform and cone–plate geometry (40 mm with a 2° angle). The method was set as follows: shear rate 100 s ^–1^, temperature gradient 25–60 °C.

The contact angle measurements used for the free surface energy calculation were performed with the cured mixtures with varying MISD content in EASO (0, 10, 30, and 50% MISD content), similar to the rheological measurements. The mixtures were cured by photoinitiation of the BAPO initiator (1% w/w in each system) and with the irradiation source (LED of 405 nm of wavelength). The liquid precursor solution was poured into a mold made of PP surface. Thin (50–250 μm) layers of cured systems were produced. The curing lasted 30 min for each mixture with 8 mW·cm^–2^ LED source power. All prepared specimens were analyzed by FT-IR to calculate their degree of cure (DC). The prepared samples exhibited contact angle measurements consisting of 10 measurements for three different liquids (eventually 30 measurements for each cured system). The liquids used were water, di-iodo methane (nonpolar liquid), and glycerol (polar substance). Once the contact angles for every liquid were measured, the free surface energy values were calculated according to the Lewis acid–base principle.

## 3 Results and Discussion

### 3.1 Production of MISD Using LLE Purification

The synthesis of MISD included the primary acylation of isosorbide with methacrylic anhydride in alkaline conditions of potassium acetate, producing a crude mixture of formed MISD and ISDMMA. The reaction mechanism of the potassium acetate catalysis has been published in our previous study.^60^ As a biobased catalyst, potassium acetate is significantly cheaper, less toxic, and more available than the most used catalyst for methacrylation, DMAP.^48^ We decided to use this catalyst due to its proven activity in our published study. Our strategy did not include any additional solvent for the reaction, and the mixture was composed of only isosorbide, methacrylic anhydride, and catalyst. The results indicated the nonselectivity of the reaction. Therefore, the purification process followed after the synthesis. The distillation of the formed secondary product—methacrylic acid—is an essential improvement in the production of MISD compared to references from the literature since it ensures the obtaining of the MAA for a potential usage and also prevents the purification process, including the washing of formed acid by neutralization solution, brine, or water. Once MAA was separated, the crude mixture was washed with a minimal amount of water twice (20 mL, one dosage) to remove any residual MAA and mainly to separate the catalyst and unreacted isosorbide from the solution. The crude mixture of MISD and ISDMMA was then mixed with water to isolate MISD. Isosorbide monomethacrylate exhibits solubility in water based on its structure containing a vacant hydroxyl group. Therefore, the extraction of MISD to the water was chosen, followed by the liquid–liquid extraction (LLE) of MISD from water to ethyl acetate, which is nonmiscible with water. Ethyl acetate was used for a more convenient solvent distillation than the water. This downstream process is unique compared to previously published isolation routes (flash chromatography/complete washing of secondary products). The obtained yields, which were weighted after the purification process, are shown in Table 1. The total amount of harvested product does not reach a quantitative yield (close to 100%) due to the combination of factors. First, methacrylic anhydride (MAAH) used for the reaction has 94% purity and contains free methacrylic acid (MAA), which does not contribute to the acylation reaction (using the particular catalyst). Also, the formation of ISDMMA requires twice the amount of acyl donor compared to MISD. Therefore, MAAH in the mixture contributes to the formation of ISDMMA and cannot produce MISD due to the deficiency. The unreacted isosorbide and catalyst were washed of the reaction mixture with the minimal amount of water (mentioned earlier). The leftover isosorbide (ISD) can be recrystallized or separated via distillation so as not to lose it and can be used further in the reaction.

**Table 1:** Isosorbide Monomethacrylate (MISD) and Secondary Products of the Reaction–Isosorbide Dimethacrylate (ISDMMA) and Methacrylic Acid (MAA) Yield Results

evaluation of the MISD synthesis			
product compound	*n* [mol]	*m* [g]	yield [%]
crude mixture (ISDMMA + MISD)	0.735	164.4	
ISDMMA	0.117	33.0	11.7%
MISD	0.618	132.4	61.8%
MAA (distilled)	0.934	81.4	93.4%

**Figure 3:**
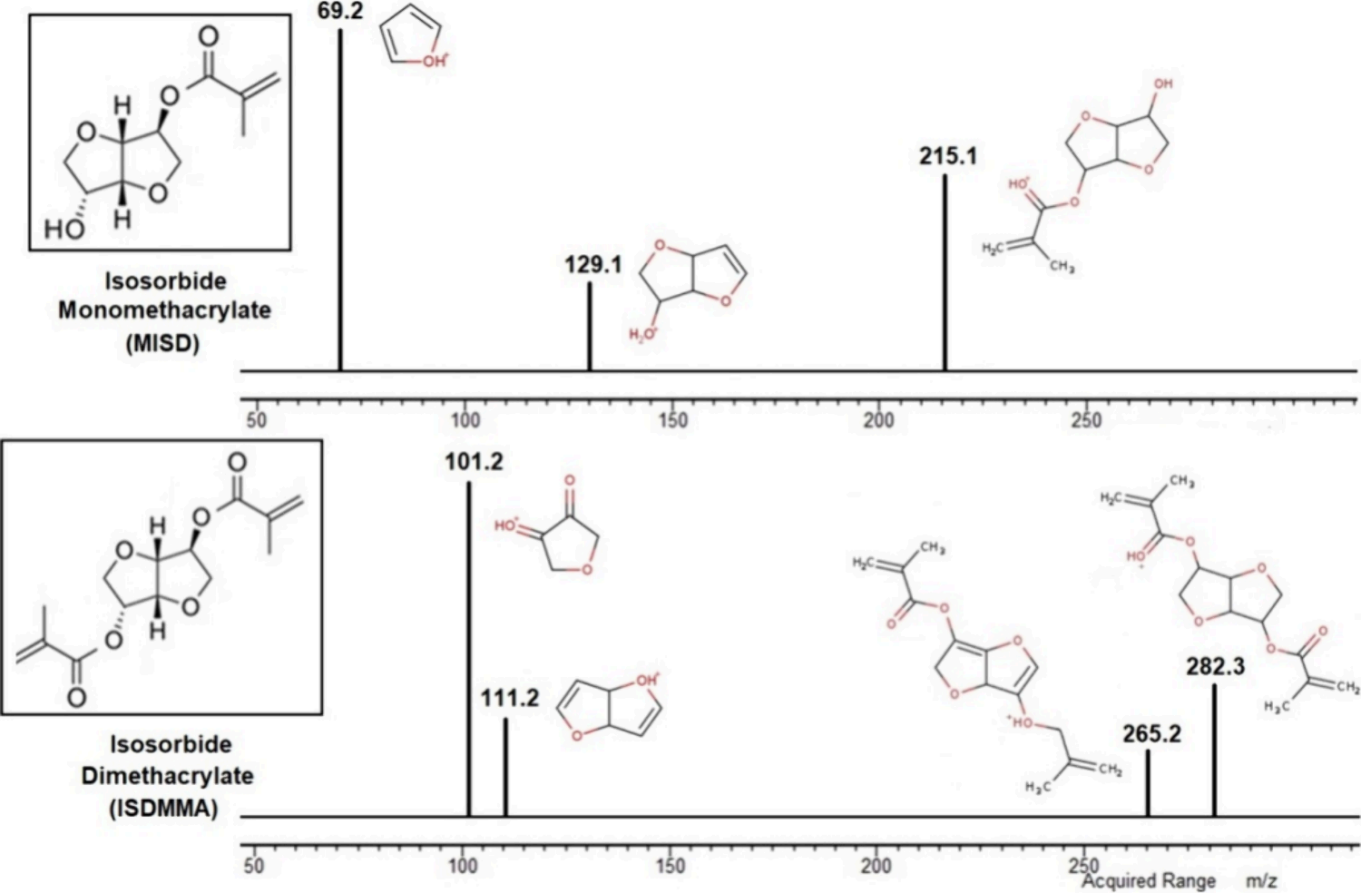
Mass spectra of synthesized isosorbide monomethacrylate (MISD) and isosorbide dimethacrylate (ISDMMA) containing the molecular peaks and particular fragments.

The structural verification included numerous analyses, including ^1^H NMR. The results of nuclear magnetic resonance measurements are summarized in the peak description in the previous chapter (chapter 2.3) and the Supplementary (Figures S1 and S2). Besides NMR analysis, ESI-MS was applied as one of the confirmation methods. The mass spectrum, in combination with other methods, verified the structures of both MISD as the main product and ISDMMA as the secondary coproduct. The spectra are shown in Figure 3, and the original data measured are also in Supplementary (Figures S3 and S4). Both MISD and ISDMMA mass spectra contained molecular peaks ([M + H]^+^ of MISD occurred as an *m*/*z* value of 215.1 and [M + H]^+^ of ISDMMA *m*/*z* value was 282.3), which confirmed the molar masses of both products. Also, other fragments were detected in the respective spectrum, verifying the structure (see Figure 3).

**Figure 4:**
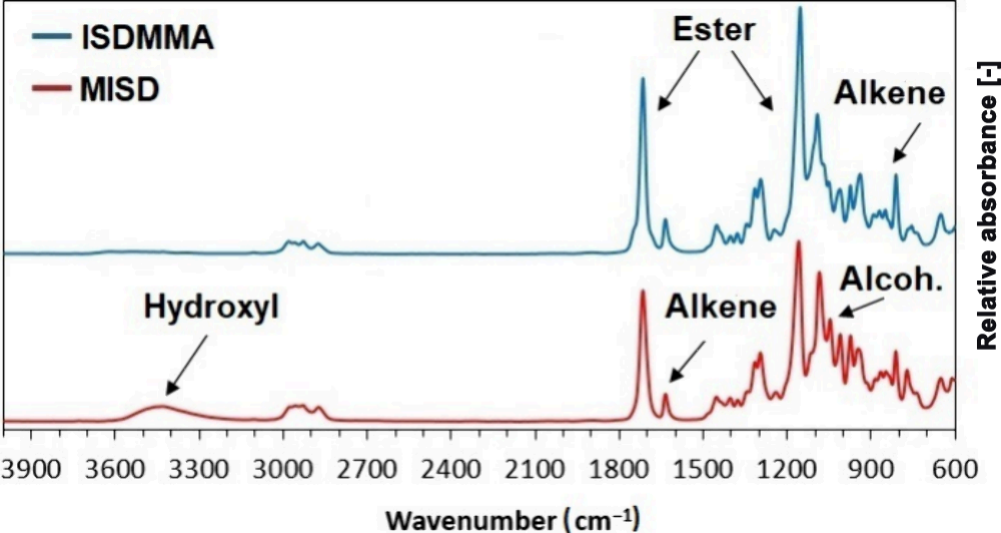
Infrared spectra of isosorbide monomethacrylate (MISD) and isosorbide dimethacrylate (ISDMMA).

Fourier-transform infrared spectroscopy (FTIR) was used among the confirmation methods to provide a crossing analysis of the formed products. The comparison of MISD and ISDMMA spectra is shown in Figure 4, and the original spectral data are a part of Supplementary Figures S5 and S6. The most noticeable difference occurs in the wavenumber interval of 3600–3300 cm^–1^ (O–H stretching) due to the absence of a vacant hydroxyl group in the structure of ISDMMA and the presence of vacant hydroxyl in MISD. Also, another verification of the existence of a hydroxyl group in MISD occurs in the interval of values of 1130–1080 cm^–1^ (C–O stretching). Both shown spectra contain signals belonging to ester functional groups within the wavenumber values of 1750–1735 cm^–1^ (C=O stretching) and 1210–1160 cm^–1^ (C–O stretching), confirming the presence of the methacrylate functional group. Subsequently, signals confirming the presence of alkene, as a part of a methacrylate group, can be found in wavenumber intervals of 1680–1660 cm^–1^ (C=C stretching) and 840–780 cm^–1^ (C=C bending).

### 3.2 Thermal Properties of Synthesized MISD

**Figure 5:**
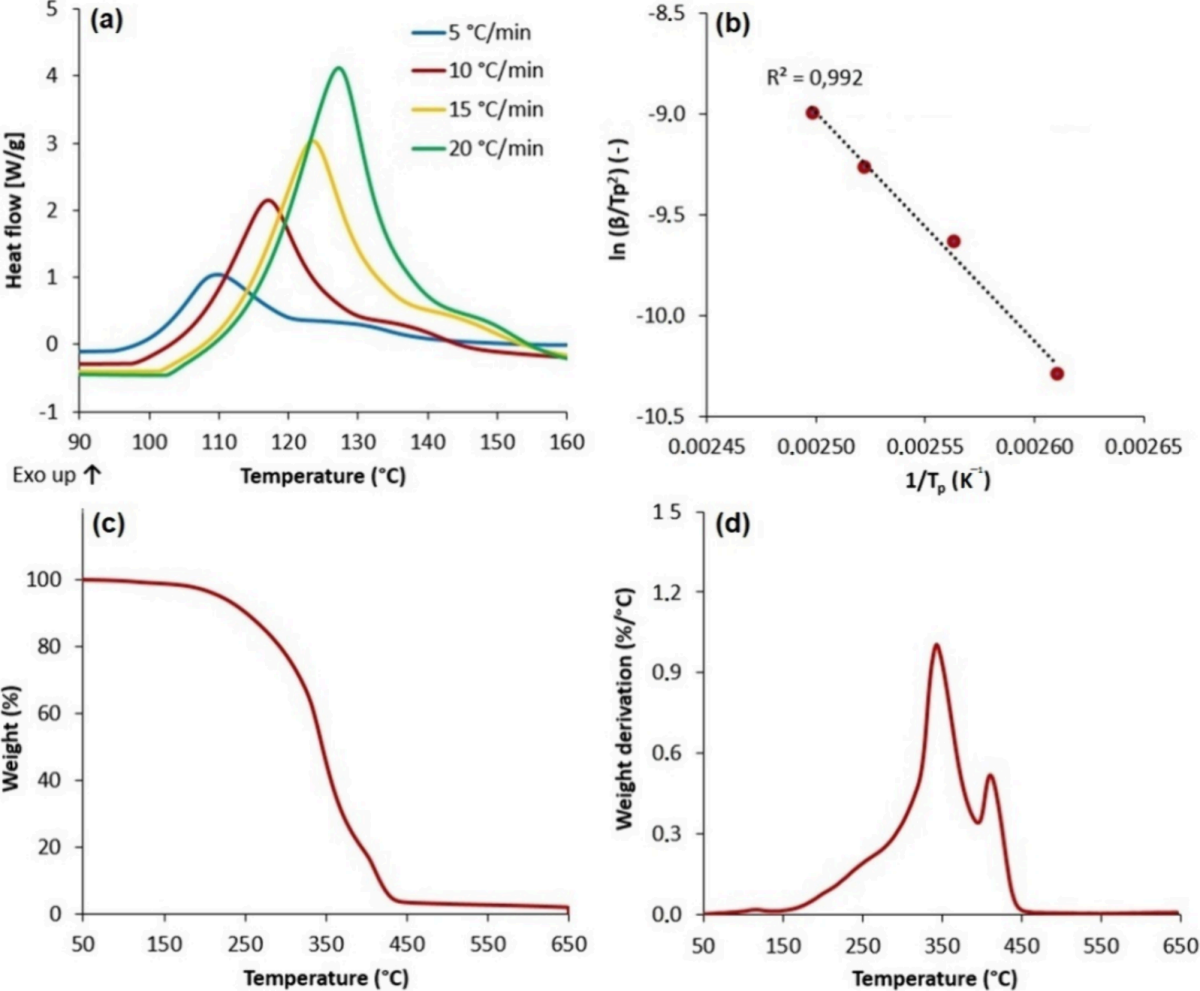
(a) DSC curves of MISD with the *tert*-butyl peroxide at different heating ramps; (b) the graphical interpretation of Kissinger’s theory; (c) TGA curve of the dependence of weight loss on the temperature; (d) the derivative TGA curve.

The curability of the synthesized MISD was confirmed via the DSC method using *tert*-butyl peroxide as a thermal initiator. This initiator has a higher initiation temperature interval (130–150 °C with a 10–1 h half-life). Therefore, the scanning temperature was set to 200 °C to observe exothermic processes during the radical reaction. Also, the mathematical description characterizing the comparable parameters of curable systems was applied. According to the literature, Kissinger’s theory defines the relationship between the heating rate of the curing process connected with the exothermic maximum and the activation energy of the process along with the pre-exponential factor, which is directly related to the reaction kinetics of the process. The equation illustrating Kissinger’s theory is given as follows:^55^
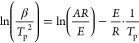
1where β stands for the heating rate (K/min); *T*_p_ represents the exothermic maximum (K); *A* exemplifies the pre-exponential factor (−); *E*_a_ is the activation energy (J/mol), and *R* is the universal gas constant (J/(mol·K)). The parameters calculated from this equation can serve as comparison values between other reactive precursors. Surely, the analogy can be considered when comparing systems that were cured by using the same initiator. Then, the exothermic maxima and calculated activation energies represent the analogical role. The calculated parameters of MISD (activation energy and pre-exponential factor) are shown in Table 2. The graphical illustrations of performed measurements and calculated Kissinger’s theory are shown in Figure 5a,b. The remaining data regarding the DSC analyses are illustrated in Supplementary (Table S1). The calculated MISD’s activation energy exhibits 94.6 kJ/mol, and the pre-exponential factor (ln(*A*) = 28.78) confirms that synthesized MISD is a reactive monomer. Both mentioned parameters can be compared to synthesized curable monomers from our previous study, which reached considerably higher values.^56^ The comparison uncovers that the reactivity of MISD is higher than those of previously introduced and described precursors.

**Table 2:** Results of DSC and TGA Analysis of Synthesized MISD

thermal properties of MISD			
DSC		TGA	
*E*_a_ (kJ/mol)	ln(*A*) (−)	*T*_max_(°C)	*T*_s_(−)
94.6	28.8	343.2	136.8

**Figure 6:**
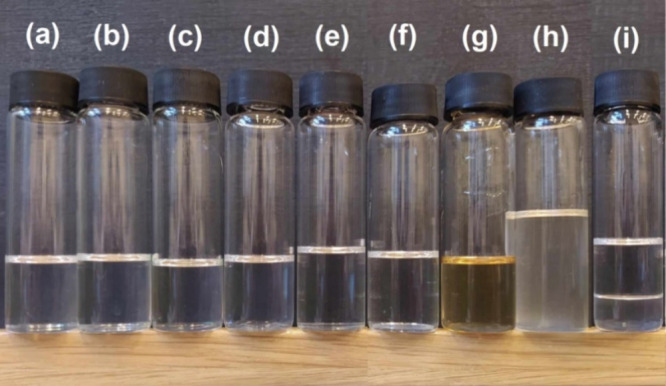
Experimental confirmation of MISD’s miscibility in different solvents. (a) Methacrylic anhydride (MAAH); (b) methacrylic acid (MAA); (c) isosorbide dimethacrylate (ISDMMA); (d) water; (e) ethanol; (f) ethyl acetate (EtAc); (g) epoxidized acrylated soybean oil (EASO); (h) isosorbide monomethacrylate solventless (MISD); (i) hexane as an inappropriate solvent for MISD.

The cured MISD was exposed to the TGA analysis to investigate the thermal degradation properties of resins composed of this precursor. The evaluation of the thermal degradation behavior summarizes the heat-resistant index (*T*_s_), which was repeatedly used in the literature for this purpose.^35,57^ The calculation of the heat-resistant index is formulated as follows:
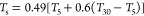
2where *T*_s_ stands for the heat-resistant index (−); parameters *T*_5_ (°C) and *T*_30_ (°C) represent the exact temperature at which 5 and 30% mass loss occur. The measured mass loss temperatures (including the temperature of the fastest thermal degradation (*T*_max_) marking the inflection point of the gravimetric curve), along with the calculated heat-resistant index of cured MISD, are shown in Table 2. The graphical illustration of the performed TGA analysis is also shown in Figure 5c,d. The calculated heat-resistant index is comparable to the previously mentioned methacrylated esters described in a previous article.^56^

### 3.3 Evaluation of the Prepared Curable Oil–MISD Mixture Properties

#### 3.3.1 Solubility of MISD

The solubility of MISD in different media was calculated according to Hansen’s theory of miscibility,^53^ which evaluates the particular summaries of the complete solubility parameter (δ). The solubility parameter consists of three distinguished components—dispersion forces, polar forces, and hydrogen bonding. The fundamental principle of calculation is detailed in the Supplementary. The complete solubility parameters were calculated for all experimentally investigated media, and according to the theoretical equations, RED values were calculated for every solvent MISD was exposed to be mixed with. The theory describes that once RED reaches a value below 1, the particular compound (MISD) is miscible with a specific solvent. The calculated RED values of all substances are summarized in Table 3. Also, the experimentally performed miscibility test results are illustrated in Figure 6. Theoretically, all chosen media should be appropriate for solution formation with MISD. Naturally, water and ethyl acetate fulfilled this prediction since these two media were used for purification after MISD synthesis. Mainly, the miscibility with the epoxidized acrylated soybean oil (EASO) was theoretically estimated and experimentally verified, which was essential for any further potential experiments of MISD’s usage as EASO rheological behavior and surface energy modifier. The MISD case concluded that the vacant hydroxyl group in the compound’s structure plays a significant role, especially in the solubility with water as a solid polar solvent. The isosorbide derivatives miscibility was investigated in previous articles.^35^ However, the solubility of pure MISD in particular has not yet been studied. The water solubility of the mixture of MISD and ISDMMA was observed in the literature.^48^ We improved the complications connected to the water solubility (lower final yields) with the addition of ethyl acetate to the LLE process to harvest the majority of our product without significant loss.

**Table 3:** Theoretical and Experimental Results of MISD’s Miscibility Investigation in Various Solvents

the miscibility of MISD			
compound	RED^a^	calculated miscibility of MISD	experimental miscibility of MISD
MISD			
MAAH	0.46	miscible	miscible
MAA	0.34	miscible	miscible
ISDMMA	0.41	miscible	miscible
water^b^	0.95	miscible	miscible
ethanol	0.15	miscible	miscible
EtAc	0.44	miscible	miscible
EASO	0.42	miscible	miscible


a RED value was calculated according to the equations described in the Supplementary.

b The solubility parameters of water were obtained from the literature.^58^

#### 3.3.2 Rheological Behavior Modifications by MISD

**Figure 7:**
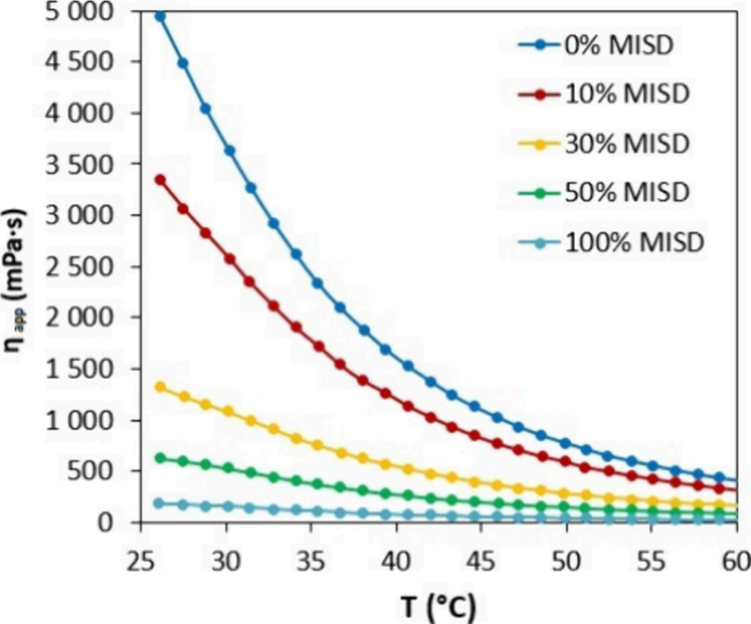
Dependences of apparent viscosity (η_app_) on increasing temperature (*T*) of MISD’s mixtures with EASO.

The rheological modification properties of MISD were investigated in the mixture with high-viscous EASO. The modified vegetable oils are vastly investigated as biobased matrices for various usage fields such as SLA 3D printing or coating applications. However, these precursors exhibit high apparent viscosity values and must be modified for specific purposes. The synthesized MISD was investigated as a curable monomer capable of incorporating cured EASO’s resin structure and decreasing the primary matrix’s apparent viscosity. The rheological dependences of apparent viscosity (η_app_) on the increasing temperature (*T*) of prepared mixtures of EASO with MISD are illustrated in Figure 7. The increasing amount of MISD in EASO decreases the apparent viscosity of the mix. The essential factor in this rheological behavior modification is the absolute miscibility of EASO in MISD, which was confirmed earlier.

The Arrhenian plot was used to mathematically evaluate rheological behavior in temperature conditions.^54^ The equation defining the connection of apparent viscosity changes with increasing temperatures describes the following equation:
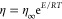
3where η stands for the dynamic viscosity (generally, in the cases of potential non-Newtonian fluid this parameter describes the apparent viscosity) (Pa·s); η_∞_ represents the “infinite-temperature” viscosity (mathematically pre-exponential factor) (Pa·s); *E* stands for the exponential constant (in the literature introduced as the flow activation energy) (J/mol); *R* is the universal gas constant (J/(mol·K)); and *T* represents the thermodynamic temperature (K). The introduced eq 3 is often reconstituted to a linear form to simplify the graphical interpretation:
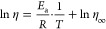
4We applied the measured results of apparent viscosity with decreasing temperature of all prepared curable mixtures, and the results are summarized in Table 4. The graphical interpretation of the Arrhenius plot is illustrated in the Supporting Information (Figure S7). The calculated values fundamentally confirm the experiments that were performed. MISD exhibits the lowest apparent viscosity and the lowest value of “infinite-temperature” viscosity and exponential factor. Therefore, MISD can be considered a suitable curable precursor for the rheological behavior modification of high-viscous matrixes such as the investigated EASO.

**Table 4:** Calculated and Summarized Parameters from Arrhenius Dependency of the Apparent Viscosity on the Temperature of MISD-Containing Mixtures

Arrhenius function parameters				
mixture	η_30°C_(mPa·s)	η_∞_(mPa·s)	*E*(kJ/(mol·K))	*R*^2^
0% MISD in EASO	3413	3.72 × 10^–7^	57.8	0.999
10% MISD in EASO	2444	6.03 × 10^–7^	55.8	0.999
30% MISD in EASO	1032	1.74 × 10^–6^	50.9	0.999
50% MISD in EASO	500	2.78 × 10^–6^	47.9	0.999
100% MISD	139	4.12 × 10^–6^	43.7	0.995

#### 3.3.3 Free Surface Energy Modification by MISD

The cured resins based on a modified vegetable oil matrix (such as EASO) tend to exhibit hydrophobic behavior. Although the hydroxyl functional groups are generated within the oil structure during the acrylic/methacrylic modification of epoxidized oils, the hydrophobic character of these materials is a consequence of the overwhelming dispersion forces in the material. Since MISD is a reactive curable compound miscible with water, the surface energy modification ability is expected when MISD becomes a part of oil’s resin site. The detailed principle of the solid state’s free surface energy calculation from the measured contact angle is described in the Supplementary. The acid–base theory^59^ of the surface energy can be applied using the following final equation for the calculation:
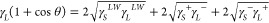
5

This equation is detailed in the Supporting Information with the whole derivation process. The parameters contributing to the total solid-state surface energy (γ_S_) are the Lifshitz–van der Waals parameter of the solid-state surface tension (γ_S_^LW^) and Lewis solid-state components, particularly the acid component (γ_S_^+^) and the alkali component (γ_S_^–^). These three parameters can be calculated when at least three particular liquid media are used for the contact angle (θ) measurements (because of the number of three unknown parameters in total). In our study, water, di-iodo methane, and glycerol were used to investigate the free surface energy of the prepared oil-based MISD-containing resins. The measured contact angles, calculated free surface energies, and determined degree of cure (DC) values are summarized in Table 5.

**Table 5:** Summarization of the MISD Containing Resins’ Free Surface Energy Values

the surface energy modification by MISD					
resin	contact angle (water)	contact angle (di-iodo methane)	contact angle (glycerol)	free surface energy^a^γ_S_(mJ/m^2^)	degree of cure^b^(DC) (%)
0% MISD in EASO	80° ± 3°	35° ± 3°	71° ± 3°	42.2	89.2
10% MISD in EASO	74° ± 3°	40° ± 5°	59° ± 3°	43.4	86.7
30% MISD in EASO	67° ± 3°	44° ± 2°	56° ± 3°	44.1	86.9
50% MISD in EASO	59° ± 3°	47° ± 1°	43° ± 3°	48.7	84.8

a The detailed free surface energy calculation is described in the Supporting Information.

b The DC values were calculated from FT-IR measurements described and are exhibited in the Supporting Information.

**Figure 8:**
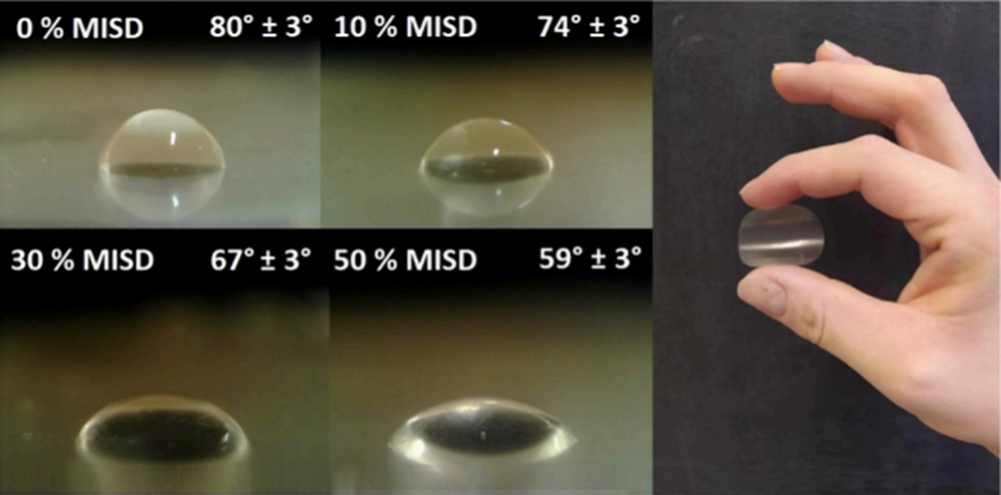
Water contact angles (θ) measured for different MISD involving EASO resins.

All measured contact angles confirmed the increased hydrophilic character of the oil-based cured resin involving MISD. Both polar liquids (water and glycerol) exhibited a decreased contact angle, indicating a higher affinity to the solid state. At the same time, the nonpolar representative (di-iodo methane) reached a higher value of the contact angle, verifying the hydrophobic character loss. Also, the degree of cure reached sufficiently high values, slightly decreasing with the MISD content in the resins. This factor might be caused by the rigid character of the isosorbide’s structure, resulting in decreased cured resin’s site molecular mobility and complicating the radical reaction. The pictures of water contact angles formed during the surface energy investigation are shown in Figure 8, along with the produced investigated material sample presentation. MISD’s surface energy modifications have not been studied, which is the novelty factor of our investigation. However, the surface energy modification properties of materials containing isosorbide derivatives were observed and published in previous work.^61^ The isosorbide derivatives incorporated into the printable materials for scaffolds exhibited the hydrophilic character similar to MISD containing oil printable resins we propose.

## 4 Conclusions

The main aim of this work was to introduce improvements in the production of isosorbide monomethacrylate (MISD). The synthesis of MISD was experimentally performed, reaching a reasonable yield of nearly 62%, higher than that in most published studies. Additionally, the catalyst used in our study, potassium acetate (CH_3_COOK), is a biobased substance that is significantly cheaper and less generally harmful than other catalysts used for MISD’s synthesis. Also, no solvent was used for the dissolution of reactants, which complicates the scale-up, and all reactants were used in equimolar quantities, which minimized the accumulation of unreacted shares. Harvesting the secondary product, methacrylic acid, which was distilled nearly quantitatively (93% yield), is the main improvement of the MISD production process in this article. Not only is the disposal process of the acid solution solved by this step, but the formed methacrylic acid can be further used for other purposes. Liquid–liquid extraction (LLE) purification ensures the scalability of our introduced MISD manufacturing process, since no chromatography is required. The purification strategy described in this work is circular since the same liquid media can be used repeatedly. The purity of the MISD that was produced confirmed multiple analyses such as ^1^H NMR, MS, or FTIR. The produced MISD was investigated by thermal analyses, DSC (the activation energy *E*_a_ reached 94.6 kJ/mol and the pre-exponential factor ln(*A*) value is 28.8), and TGA (the temperature of the maximal thermal degradation *T*_max_ is 343.2 °C and the heat-resistant index of cured MISD *T*_s_ is 136.8), which verified MISD’s curability and the specific thermal stability of the cured resin. Combining MISD with a nonpolar resin precursor, epoxidized acrylated soybean oil (EASO), was investigated from the miscibility, rheological, and surface energy perspective. It was found that EASO is miscible with MISD, although this synthesized molecule exhibits solubility in water (MISD possesses a strong polar character). Subsequently, the apparent viscosity of EASO (η_30°C_ = 3413 mPa·s) decreased with the rising MISD content (η_30°C_ of EASO containing 50 w% of MISD is 500 mPa·s), and the surface energy of MISD-containing resins reached higher values (increased from 42.2 mJ/m^2^ (100% EASO) to 48.7 mJ/m^2^ (50% EASO with 50% MISD)). These results uncover the MISD’s potential to serve as a viscosity and polarity modifier of curable vegetable oils.

## Supporting Information Available

Additional supportive material regarding the article’s studies; particular NMR, MS, and FTIR spectra, and other data are acquired from DSC and TGA analyses; detailed description of the miscibility and surface energy calculation can be found in the SI, along with the additional results and graphs from the miscibility, rheological, and surface energy determination study (PDF)
