# Supplementary material for: Improvements in the Production of Isosorbide Monomethacrylate Using a Biobased Catalyst and Liquid–Liquid Extraction Isolation for Modifications of Oil-Based Resins
Source: ACS Omega. 2024 May 31;9(23):24728–38. doi: 10.1021/acsomega.4c01275 (PMC11171093; doi:10.1021/acsomega.4c01275)
Supplement: Supplementary file 1 — ao4c01275_si_001.pdf [file ao4c01275_si_001.pdf]

Improvements in the production of isosorbide monomethacrylate using a biobased catalyst and liquid-liquid extraction isolation for modifications of oil-based resins

Vojtěch Jašek[a*], Jan Fučík[b], Veronika Melčová[a], Radek Přikryl[a], Silvestr Figalla[a]

a Institute of Materials Chemistry, Faculty of Chemistry, Brno University of Technology, 61200 Brno, Czech Republic.

b Institute of Environmental Chemistry, Faculty of Chemistry, Brno University of Technology, 612 00 Brno, Czech Republic

*corresponding author: xcjasekv@vutbr.cz

Supporting Information

1. Structural characterization spectra and description

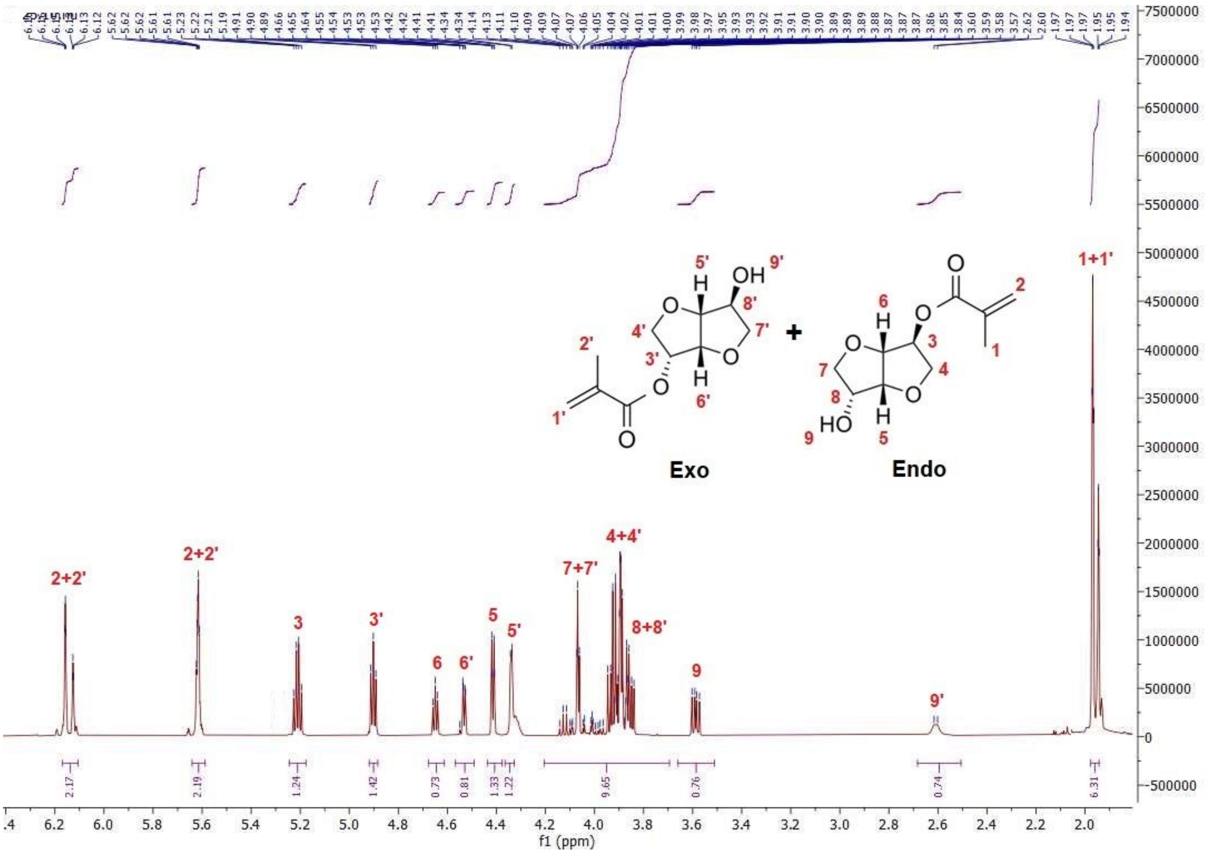

Figure S1. **^{1}H NMR spectrum of Isosorbide monomethacrylate (MISD)** ($CDCl_3$, 500 MHz): δ (ppm) 6.17-6.11(dt, *J* = 15.6, 1.3 Hz, 2H); 5.64–5.59(dt, *J* = 3.1, 1.5 Hz, 2H); 5.24–5.18 (m, 1H); 4.92–4.88 (t, *J* = 5.1 Hz, 1H); 4.68–4.61(t, *J* = 4.8 Hz, 1H); 4.57–4.49 (dd, *J* = 4.4, 1.2 Hz, 1H); 4.44–4.38 (dd, *J* = 4.6, 1.1 Hz, 1H); 4.37–3.33 (d, *J* = 3.2 Hz, 1H); 4.21–3.69 (m, 10H); 3.66–3.51 (dd, *J* = 9.5, 6.0 Hz, 1H); 2.68–2.51 (d, *J* = 7.0 Hz, 1H); 1.98–1.94 (m, 6H).

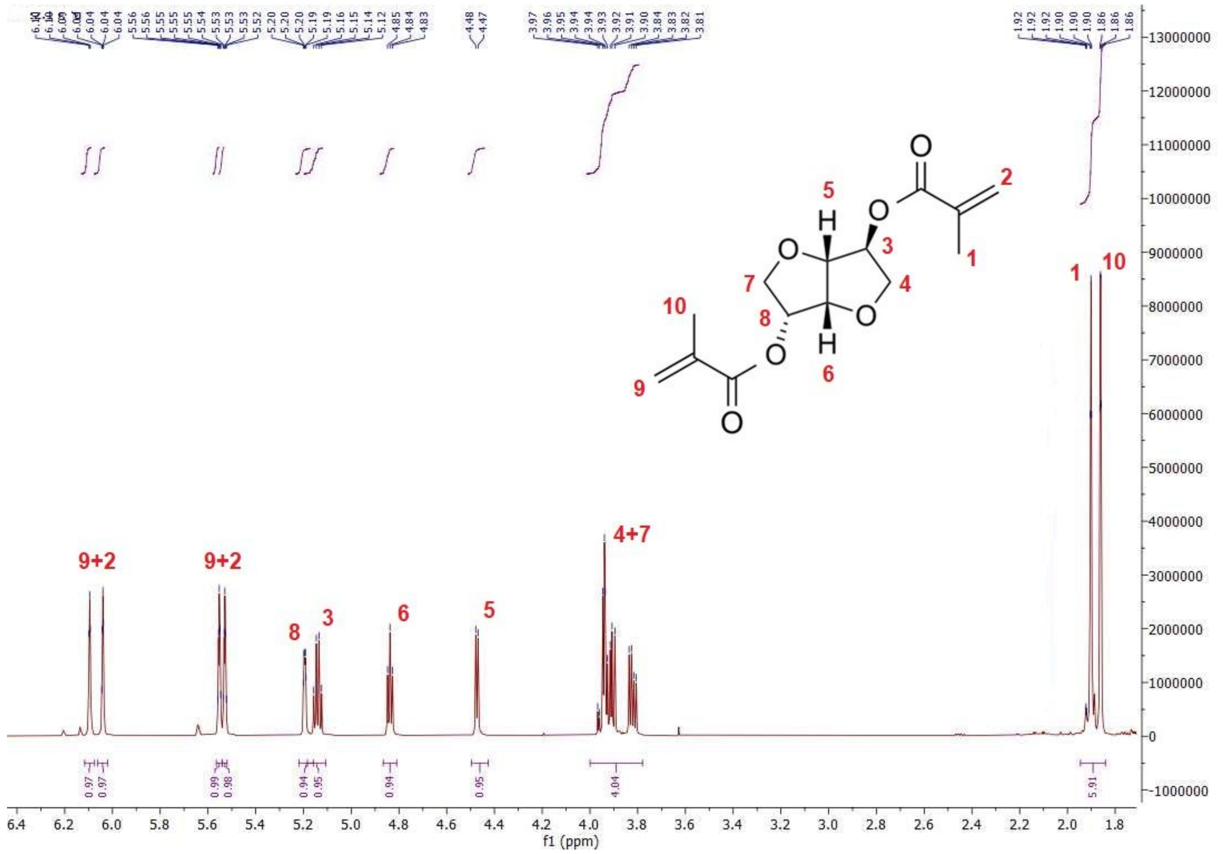

Figure S2. ^{1}H NMR spectrum of Isosorbide dimethacrylate (ISDMMA) ($CDCl_3$, 500 MHz): δ (ppm) 6.12-6.08 (t, *J* = 1.3, 1.3 Hz, 1H); 6.07–6.02 (q, *J* = 1.2, 1.1, 1.1 Hz, 1H); 5.58–5.51 (dp, *J* = 1.7, 1.6, 1.6, 1.6, 1.6, Hz, 2H); 5.22–5.16 (q, *J* = 2.9, 1.2, 1.2 Hz,1H); 5.18–5.11 (q, *J* = 5.5, 5.5, 5.5 Hz, 1H); 4.87–4.81 (t, *J* = 5.1, 5.1 Hz, 1H); 4.50–4.43 (d, *J* = 4.7 Hz, 1H); 4.00–3.77 (m, 4H); 1.95–1.84 (dt, *J* = 20.0, 1.3, 1.3 Hz, 6H).

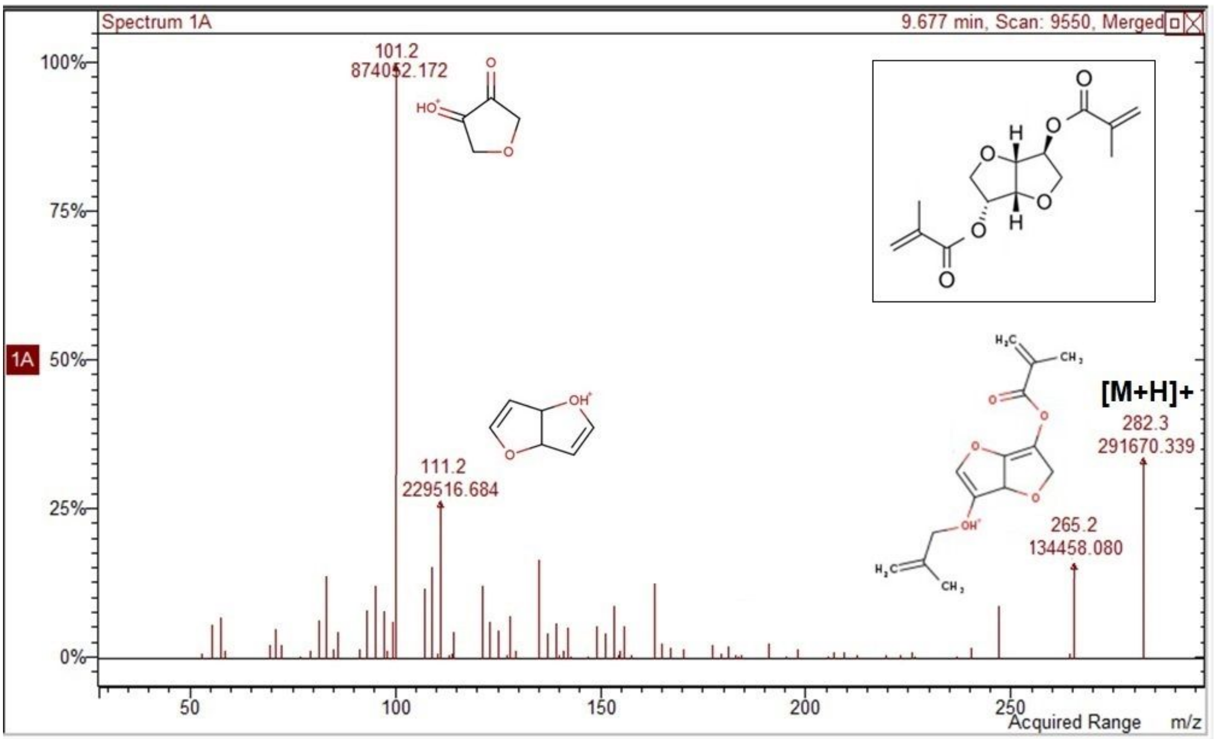

Figure S3. The MS/MS spectrum of isosorbide dimethacrylate (ISDMMA).

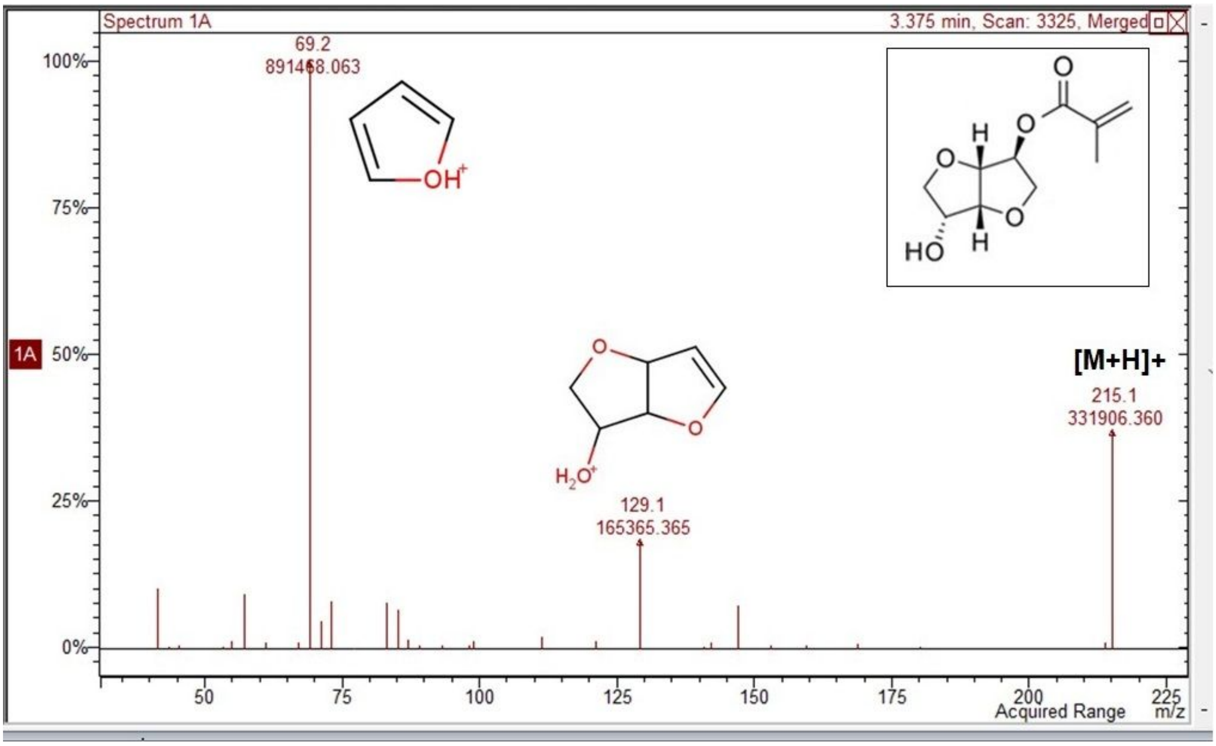

Figure S4. The MS/MS spectrum of isosorbide monomethacrylate (MISD).

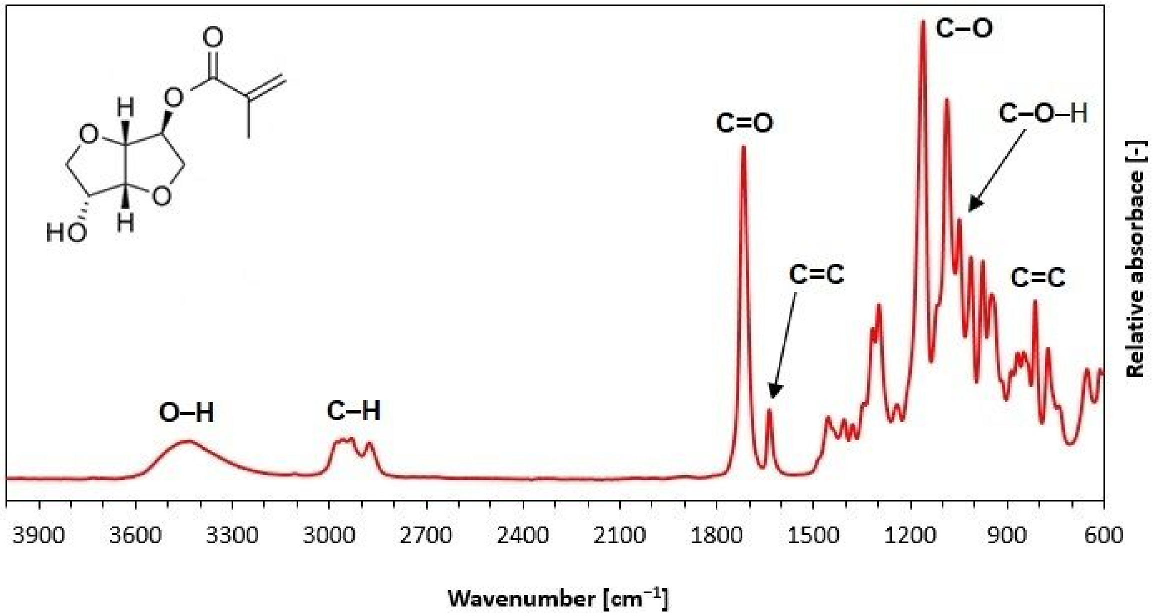

Figure S5. The FTIR spectrum of isosorbide monomethacrylate (MISD).

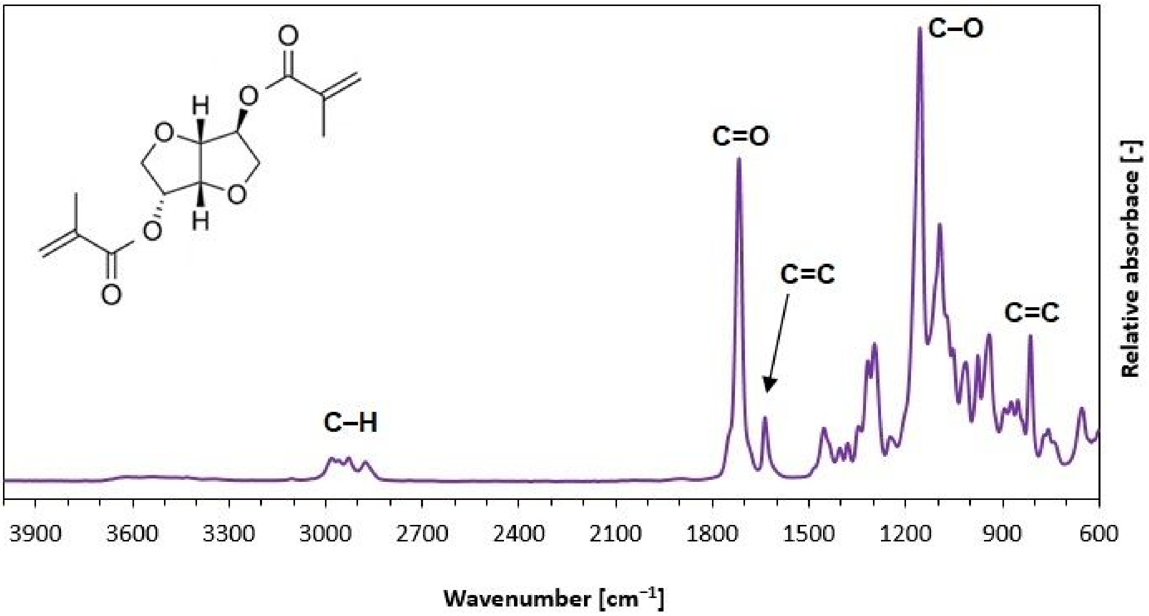

Figure S6. The FTIR spectrum of isosorbide dimethacrylate (ISDMMA).

2. Additional data regarding the thermal characterization of MISD

Table S1. The measured and calculated parameters connected to the thermal analysis of MISD.

Thermal properties of MISD						
DSC						
T_p (K)						
5 K/min	10 K/min	15 K/min	20 K/min	E (kJ/mol)	ln(A) (-)	R^2
383.11	390.23	396.56	400.33	94.6	28.80	0.992
TGA						
T_5 (°C)		T_{30} (°C)		T_{max} (°C)		T_s (-)
219.3		319.0		343.2		136.8

3. Oil-MISD properties supplementary information

3.1 MISD miscibility calculation

The theoretical miscibility of MISD was calculated according to Hansen's theory [1]. The complete solubility parameter (δ) consists of three particular square summaries: dispersion forces (δ_d), polar forces (δ_p), and hydrogen bonding (δ_h). The equation evaluating the calculation of the complete solubility parameter is formulated as follows:

$$\delta^2 = {\delta_d}^2 + {\delta_p}^2 + {\delta_h}^2, \tag{S1}$$

where all parameters defined above posses unit (J/mol). This individual intermolecular forces components' energy values can be found in Hansen's book [1]. Once the complete solubility parameters of all compounds are calculated, the "distance" solubility parameter (R_a) can be determined from the followed equation:

$$(R_a)^2 = 4(\delta_{d2} - \delta_{d1})^2 + (\delta_{p2} - \delta_{p1})^2 + (\delta_{h2} - \delta_{h1})^2, \tag{S2}$$

where the first indexed partial solubility parameter (δd1, δp1, δh1) belongs to the mixed compound (MISD particularly), and the second (δd2, δp2, δh2) describes the intermolecular forces exhibiting the solution media. When the "distance" solubility parameter of each MISD mixture solvent is calculated, the *RED* value can be reached by the following equation:

$$RED = \frac{R_a}{R_0}, \quad \text{(S3)}$$

where R_0 stands for the MISD's complete solubility parameter (δ). The *RED* value theoretically determines the miscibility of the particular compound (MISD) in any considered solvent. When RED reaches a value below 1, the solvent and compound form an analytical solution; a value above 1 indicates the miscibility inability. All determined, calculated, and summarized parameters describing the solubility of MISD are written in Table S2.

Table S2. The calculated parameters used for the theoretical RED miscibility value determination of MISD.

Compound	Dispersion forces δ_d [MJ/m^3]$^{1/2}$	Polar (Keesom) forces δ_p [MJ/m^3]$^{1/2}$	Hydrogen bonding forces δ_h [MJ/m^3]$^{1/2}$	Complete solubility parameter δ_h [MJ/m^3]$^{1/2}$	R_a	*RED*
MISD	16.30	5.72	19.71	26.21	/	/
MAAH	16.51	6.13	7.77	19.25	11.95	0.46
MAA	16.74	4.95	10.86	20.56	8.93	0.34
ISDMMA	17.47	3.91	9.35	20.19	10.78	0.41
Water[a]	15.50	16.00	42.30	47.81	24.87	0.95
Ethanol	15.08	8.38	18.30	25.15	3.88	0.15
EtAc	15.36	5.02	8.47	18.24	11.42	0.44
EASO	15.91	2.71	9.16	18.55	11.00	0.42

[a]The solubility parameters of water were obtained from the literature [2]

3.2 Rheological modification by MISD

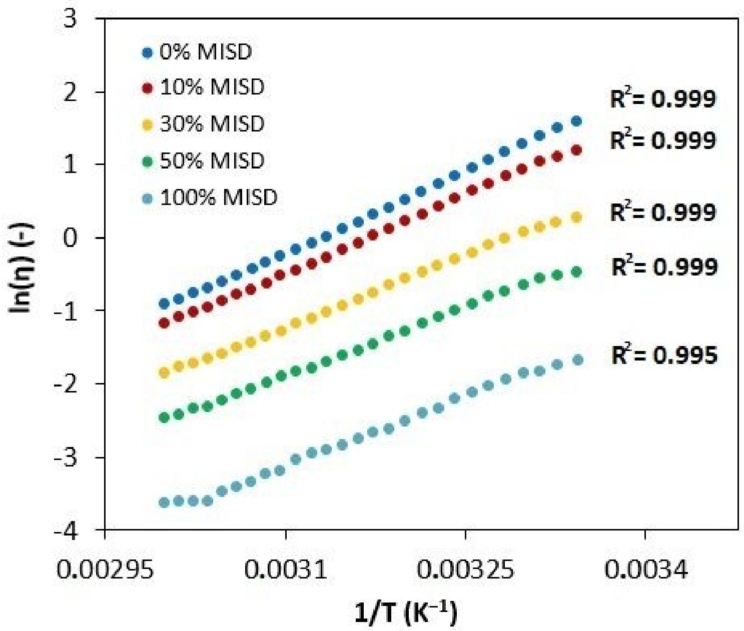

Figure S7. The graphical interpretation of Arrhenius law applied for MISD containing mixtures.

3.3 Surface energy modification by MISD

The surface energy of a solid surface (γ_S) (J/m^2) can be calculated according to Young's equation (4), which defines the solid-liquid and liquid-solid surface free energy (γ_{SL}) (J/m^2) consisting of two energetic summaries: the polarity forces (γ^P) and the dispersion forces (γ^d) (see equation (5)). Also, the surface tension of the liquid phase is defined (γ_L) (J/m^2). The Young's equation is defined as follows:

$$\gamma_S = \gamma_{SL} + \gamma_L \cos\theta \tag{S4}$$

$$\gamma = \gamma^P + \gamma^d, \tag{S5}$$

where θ stands for the contact angle of a particular liquid (°). Used acid-base theory for the calculation of the free surface energy of the solid-state (γ_S) introduces the Lifshitz-van der Waals component (γ^{LW}), which substitutes the dispersion forces parameter (γ^d). Also, the polar forces parameter (γ^P) is changed, and the Lewis components are defined as acid component (γ^+) and alkali component (γ^-). The solid-liquid surface energy is modified using the parameters of the converted force as follows:

$$\gamma_{SL} = \gamma_S + \gamma_L - 2\sqrt{\gamma_S{}^{LW}\gamma_L{}^{LW}} - 2\sqrt{\gamma_S{}^{+}\gamma_L{}^{-}} - 2\sqrt{\gamma_S{}^{-}\gamma_L{}^{+}}, \tag{S6}$$

which means that γ_{SL} can be calculated as a geometric average function of force components (for the acid-base theory, the substituted force components). When Young's equation (4) is combined with the calculation principle of γ_{SL}, the final equation for the free surface energy can be formed:

$$\gamma_L(1 + \cos\theta) = 2\sqrt{\gamma_S{}^{LW}\gamma_L{}^{LW}} + 2\sqrt{\gamma_S{}^{+}\gamma_L{}^{-}} + 2\sqrt{\gamma_S{}^{-}\gamma_L{}^{+}} \tag{S7}$$

Since the parameters contact angle (θ), liquid surface tension (γ_L), Lifshitz-van der Waals parameter of the liquid surface tension ($\gamma_L{}^{LW}$), and both Lewis components of liquid ($\gamma_L{}^+$ and $\gamma_L{}^-$) can be measured (or calculated), minimal three different liquids need to be measured to calculate the remaining parameters ($\gamma_S{}^{LW}$, $\gamma_S{}^+$ and $\gamma_S{}^-$). The complete solid-state free surface energy was calculated using the available online calculator from the measured contact angles [4]. The principle of the free surface calculation, including the particular parameters' values, was obtained from the literature [3].

Table S3. Surface free energy parameters of control liquid (mJ/m^2) [3].

Types of liquid	γ_L	γ_L^d	γ_L^P	γ_L^{LW}	γ_L^{AB}	γ_L^+	γ_L^-
Deionized water	72.80	21.80	51.00	21.80	51.00	25.50	25.50
Diiodomethane	50.80	50.80	0.00	50.80	0.00	0.00	0.00
Formamide	58.00	39.00	19.00	39.00	19.00	2.28	39.60
Glycerol	64.00	34.00	30.00	34.00	30.00	3.90	57.40

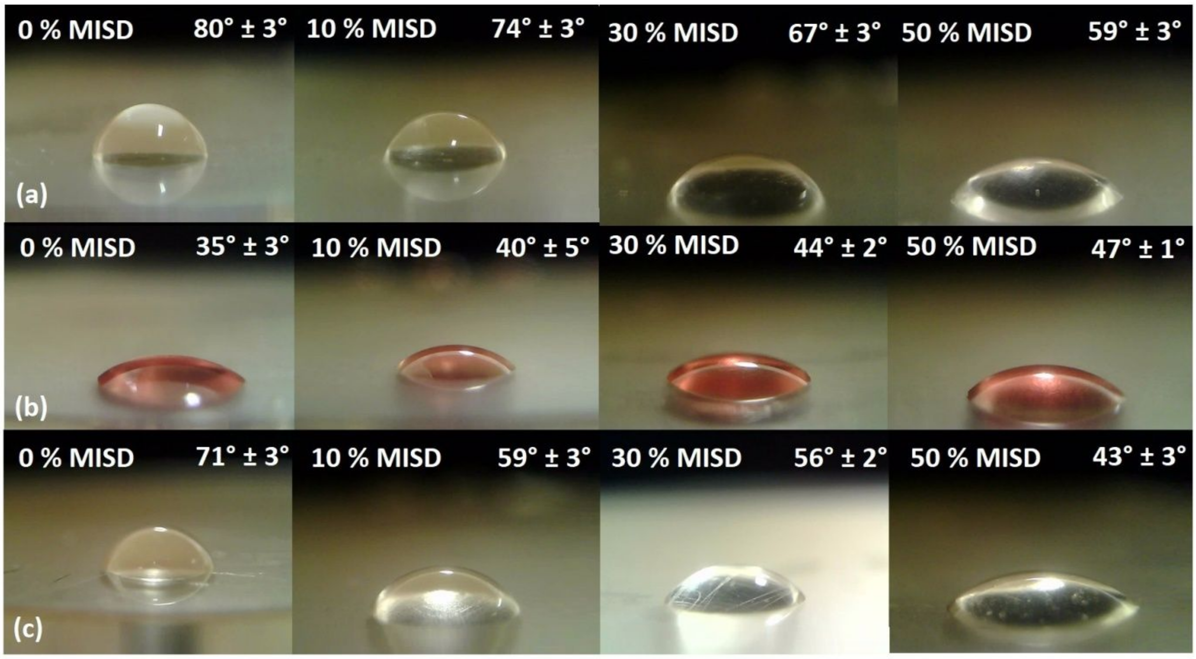

Figure S8. The photographic documentation of the contact angle's measurements of prepared MISD-containing resins.

The degree of cure (DC) of prepared resins can be calculated by the FT-IR measurement method as follows:

$$DC = \left(1 - \frac{\left(\frac{A_{C=C}}{A_{C=O}}\right)_{cured}}{\left(\frac{A_{C=C}}{A_{C=O}}\right)_{uncured}}\right) \times 100, \quad \text{(S8)}$$

where DC is the degree of cure (%); $A_{C=C}$ represents the value of the vinyl C=C stretching signal integral area (-) and $A_{C=O}$ refers to the integral area of C=O stretching signal (-).

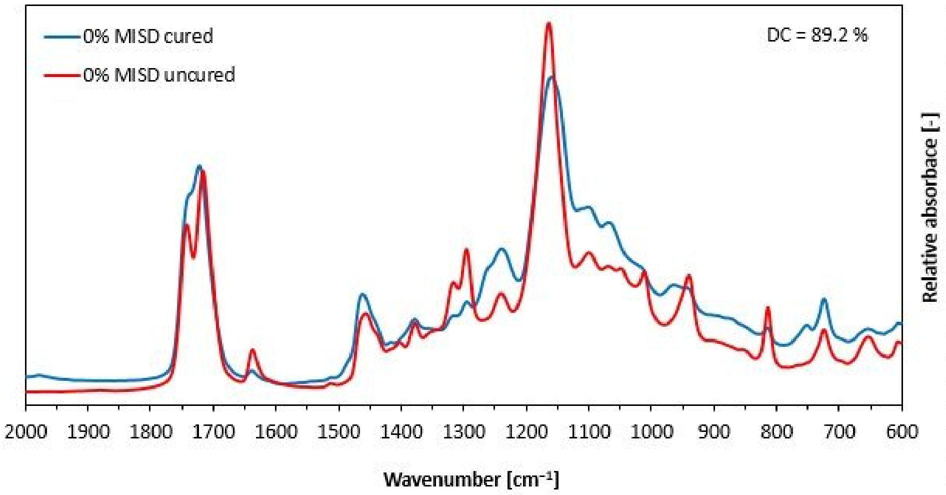

Figure S9. The degree of cure (DC) of EASO resin containing 0% of MISD measured by FT-IR.

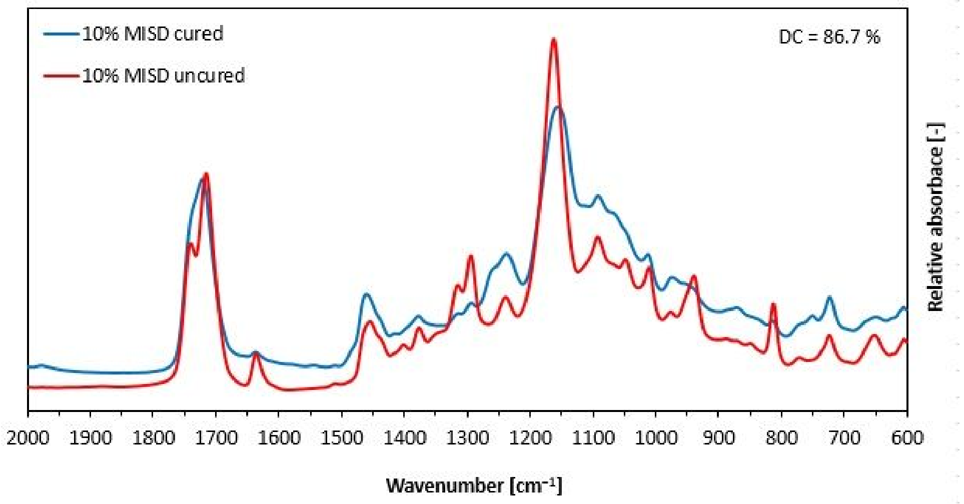

Figure S10. The degree of cure (DC) of EASO resin containing 10% of MISD measured by FT-IR.

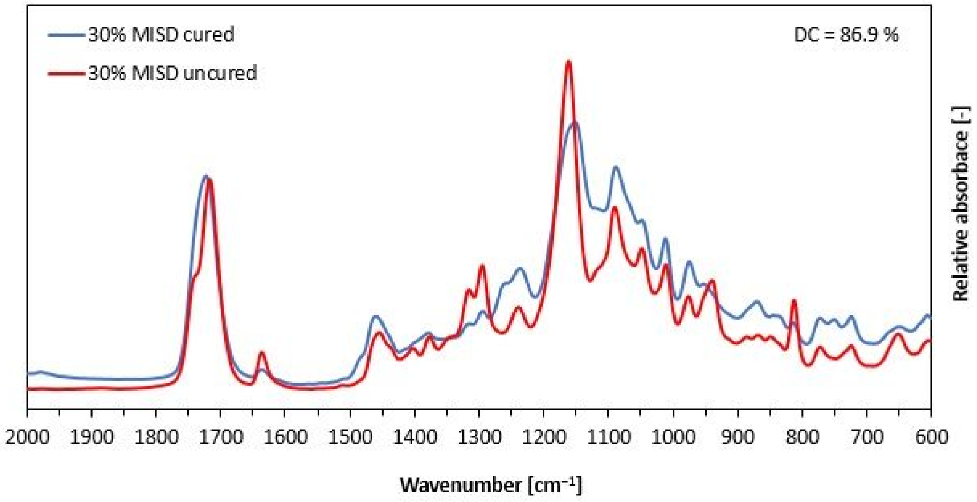

Figure S11. The degree of cure (DC) of EASO resin containing 30% of MISD measured by FT-IR.

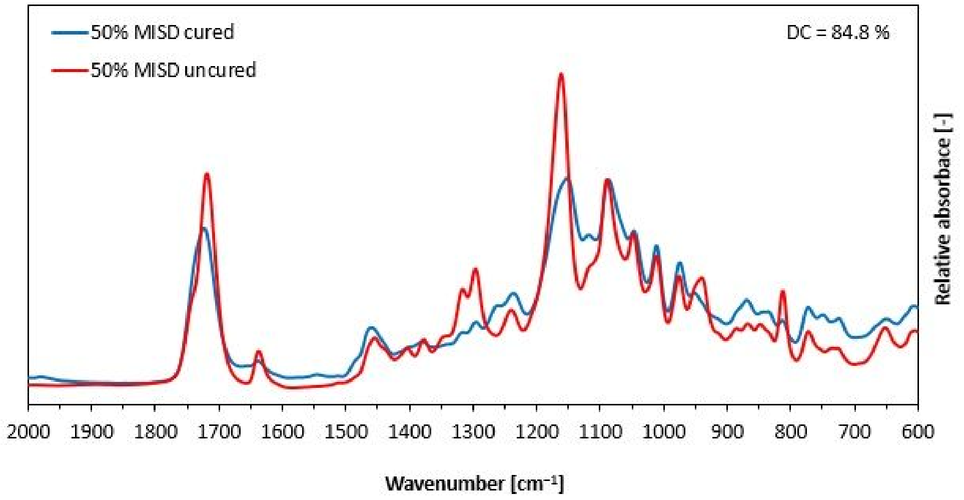

Figure S12. The degree of cure (DC) of EASO resin containing 50% of MISD measured by FT-IR.

4. References

1 Hansen, C. M. (2007). *Hansen solubility parameters: a user's handbook*. CRC press.

2 Date, A. A.; Srivastava, D.; Nagarsenker, M. S.; Mulherkar, R.; Panicker, L.; Aswal, V.; Hassan, P. A.; Steiniger, F.; Thamm, J.; Fahr, A. Lecithin-Based Novel Cationic Nanocarriers (Leciplex) I: Fabrication, Characterization And Evaluation. *Nanomedicine* **2011**, *6* (8), 1309-1325. https://doi.org/10.2217/nnm.11.38.

3 Wang, C.; Yu, X.; Smith, L.; Wang, G.; Cheng, H.; Zhang, S. Interfacial Properties Of Bamboo Fiber-Reinforced High-Density Polyethylene Composites By Different Methods For Adding Nano Calcium Carbonate. *Polymers* **2017**, *9* (11). https://doi.org/10.3390/polym9110587.

4 SEC. (n.d.). https://www.stevenabbott.co.uk/abbottapps/SEC/index.html
